# Calcium Regulation of Connexin Hemichannels

**DOI:** 10.3390/ijms25126594

**Published:** 2024-06-15

**Authors:** Erva Bayraktar, Diego Lopez-Pigozzi, Mario Bortolozzi

**Affiliations:** 1 Veneto Institute of Molecular Medicine (VIMM), Via Orus 2, 35129 Padova, Italy; 2 Department of Physics and Astronomy “G. Galilei”, University of Padua, Via Marzolo 8, 35131 Padova, Italy; 3 Institute of Endocrinology and Oncology “Gaetano Salvatore” (IEOS-CNR), Via Pietro Castellino 111, 80131 Napoli, Italy

**Keywords:** connexin, connexon, hemichannel, gap junction, calcium, sensitivity to Ca^2+^, voltage, extracellular and intracellular gating, ATP release, paracrine signalling, HC dysfunction, pathological mutations

## Abstract

Connexin hemichannels (HCs) expressed at the plasma membrane of mammalian cells are of paramount importance for intercellular communication. In physiological conditions, HCs can form gap junction (GJ) channels, providing a direct diffusive path between neighbouring cells. In addition, unpaired HCs provide conduits for the exchange of solutes between the cytoplasm and the extracellular milieu, including messenger molecules involved in paracrine signalling. The synergistic action of membrane potential and Ca^2+^ ions controls the gating of the large and relatively unselective pore of connexin HCs. The four orders of magnitude difference in gating sensitivity to the extracellular ([Ca^2+^]_e_) and the cytosolic ([Ca^2+^]_c_) Ca^2+^ concentrations suggests that at least two different Ca^2+^ sensors may exist. While [Ca^2+^]_e_ acts as a spatial modulator of the HC opening, which is most likely dependent on the cell layer, compartment, and organ, [Ca^2+^]_c_ triggers HC opening and the release of extracellular bursts of messenger molecules. Such molecules include ATP, cAMP, glutamate, NAD^+^, glutathione, D-serine, and prostaglandins. Lost or abnormal HC regulation by Ca^2+^ has been associated with several diseases, including deafness, keratitis ichthyosis, palmoplantar keratoderma, Charcot–Marie–Tooth neuropathy, oculodentodigital dysplasia, and congenital cataracts. The fact that both an increased and a decreased Ca^2+^ sensitivity has been linked to pathological conditions suggests that Ca^2+^ in healthy cells finely tunes the normal HC function. Overall, further investigation is needed to clarify the structural and chemical modifications of connexin HCs during [Ca^2+^]_e_ and [Ca^2+^]_c_ variations. A molecular model that accounts for changes in both Ca^2+^ and the transmembrane voltage will undoubtedly enhance our interpretation of the experimental results and pave the way for developing therapeutic compounds targeting specific HC dysfunctions.

## 1. Introduction

Connexins are a family of tetraspan membrane proteins encoded by 21 genes in humans [1]. The oligomerization of different connexin isoforms into hexameric structures, called connexons or hemichannels (HCs), usually occurs during the transition from the rough endoplasmic reticulum to the Golgi apparatus [2]. Only a fraction of the total connexin expressed by the cell reaches the plasma membrane [3,4], where two HCs belonging to adjacent membranes can dock end–to–end to form a junctional channel. Tens to thousands of junctional channels can line up in a dense hexagonal pattern called a gap junction (GJ) [5]. Various compounds with a molecular mass of up to approximately 1 kDa, including water molecules, ions, second messengers, amino acids, nucleotides, and glucose, can be exchanged by passive diffusion through GJ channels that connect the cytoplasms of neighbouring cells or even different regions of the same cell, as in the case of the myelin sheath [6]. While connexin HCs are normally closed at rest, they can open under physiological conditions, thus allowing sustained ion fluxes and permeation of messenger molecules [7].

**Figure 1:**
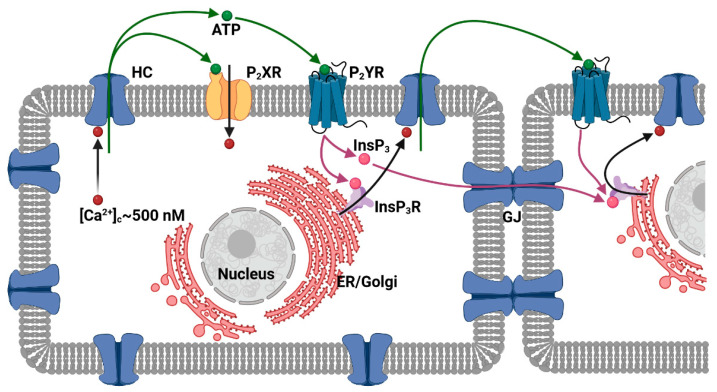
ATP signalling mediated by connexin HCs. Typically, a local submicromolar increase in the cytosolic Ca^2+^ concentration ([Ca^2+^]_c_) triggers ATP release by connexin HCs of the same cell. The ATP molecules diffuse extracellularly and can activate ATP-gated P2X ionotropic receptors and G-protein-coupled P2Y receptors. The latter trigger a canonical transduction cascade mediated by the second messenger inositol 1,4,5-trisphosphate (InsP_3_). InsP_3_ binds to and opens its receptor (InsP_3_R) that in turn releases Ca^2+^ from the endoplasmic reticulum (ER) or the Golgi apparatus. The ensuing increase in [Ca^2+^]_c_ promotes further HC openings, resulting in the propagation of a Ca^2+^ wave sustained by an ATP-induced ATP release mechanism. InsP_3_ can also diffuse through GJs, supporting the Ca^2+^ wave propagation.

Adenosine triphosphate (ATP) is a fundamental signalling molecule that can be released from the cytosol by connexin HCs, including those formed by connexin 26 (Cx26) [8,9], Cx30 [10,11], Cx30.2/Cx31.3 [12], Cx32 [13], Cx36 [14], Cx38 [15], Cx40 [16], and Cx43 [17,18]. The ATP released through connexin HCs may promote cytosolic Ca^2+^ oscillations and intercellular Ca^2+^ wave propagation by the activation of purinergic receptors [16,17,19,20,21,22] (Figure 1). Other key messenger molecules released by connexin HCs include cyclic adenosine monophosphate (cAMP) [23], glutamate [24,25], oxidized nicotinamide adenine dinucleotide (NAD^+^) [26,27], glutathione [28], D-serine [29], and prostaglandins [30,31]. These molecules can mediate autocrine and paracrine processes that regulate the development, homeostasis, function, and regeneration of several organs.

Connexin HCs have been found to be involved in cell proliferation [32], cell–cell adhesion [33], light processing by the retina [34], metabolic homeostasis, and transparency of the lens [35,36]. They are also involved in hearing maturation and function [37,38], bone growth, remodelling and protection against oxidative stress [39,40,41,42], isosmotic regulation of cell volume [43], salt and water reabsorption in the renal tubule [11], blood–brain barrier permeability [44], and the development and functionality of the peripheral and central nervous system [29,45,46,47,48,49,50]. Connexin HCs also play a role in autophagy [51], tumour growth [52], mitochondrial permeability in cardiac cells [53,54], atrioventricular conduction [55,56], vessel contractility [57], and epidermal barrier homeostasis [58].

The opening and closure of connexin HCs are regulated by several physiological parameters, such as transmembrane voltage [59,60], cell redox potential [61], phosphorylation [62,63], membrane mechanical stretch [64,65], amino sulfonates [66], CO_2_ [67], pH [68,69], and cations [70,71], including extracellular [72,73] and intracellular Ca^2+^ [13,74,75].

The complex interaction of Ca^2+^ with connexin HCs is strictly interconnected with many biological functions accomplished by this ion in the cell. This review focuses on HC regulation by Ca^2+^, one of the most important factors controlling molecular fluxes through connexin HCs in health and disease.

## 2. Extracellular Ca^2+^ Regulation of Connexin HCs

In humans, the extracellular concentration of Ca^2+^ ([Ca^2+^]_e_) is found in a narrow range of 1.1–1.4 mM in most organs [76], with the notable exception of the cochlear endolymph [77]. A reduction in [Ca^2+^]_e_ to hundreds or tens of micromolars typically causes a marked increase in the amplitude of HC currents, thus shifting their activation to more negative potentials and altering the activation and deactivation kinetics [73]. Low values of [Ca^2+^]_e_ increase the activity of most, if not all, connexin HCs, including Cx26 [78], Cx30 [60], Cx30.2/Cx31.3 [12], Cx32 [72], Cx37 [79], Cx39 [80], Cx40 [81], Cx43 [63], Cx45 [82], Cx46 [83], and Cx50 [84] (Table 1). It is important to note that connexin HCs cannot be considered as regulators of [Ca^2+^]_e_, since their opening does not activate any feedback mechanism for [Ca^2+^]_e_ adjustment. Instead, [Ca^2+^]_e_ variations modulate the HC opening probability, which is key for several downstream physiological mechanisms.

In the intact central nervous system, [Ca^2+^]_e_ can be reduced from 1.2–1.4 mM to less than 0.7 mM during periods of intense neuronal activity, a phenomenon that activates purinergic feedback signalling from astrocytes to interneurons mediated by Cx43 HCs [86]. In non-sensory cells of the cochlea, the low endolymphatic [Ca^2+^]_e_ (20–30 μM [77]) biases Cx26/Cx30 HCs towards the open state, thus altering the ATP-dependent intercellular Ca^2+^ signalling that is required for refinement of afferent innervations of outer hair cells [87]. In the mammalian epidermis, a characteristic [Ca^2+^]_e_ gradient between lower and upper layers plays a crucial role in the processes of keratinocyte differentiation and formation of the epidermal permeability barrier [88].

Specific information about the dependency of the HC gating on [Ca^2+^]_e_ is available for several connexin isoforms, as detailed in the following.

**Cx26:** Atomic force microscopy (AFM) experiments performed in isolated membranes of HeLa cells expressing rat Cx26 (rCx26) showed a reduction in the HC extracellular inner diameter from 1.3 to 0.5 nm by adding 0.5 mM Ca^2+^ to the [Ca^2+^]_e_-free solution [89]. In *Xenopus* oocytes expressing human Cx26 (hCx26), the HC current dependent on [Ca^2+^]_e_ ranged from a maximum at 0.01 mM [Ca^2+^]_e_ to a minimum value at 10 mM [Ca^2+^]_e_, with an EC50 around 0.25 mM [78]. Additionally, lowering [Ca^2+^]_e_ from 0.75 mM to 0.1 mM increased the HC current by approximately 85% at +40 mV, while increasing [Ca^2+^]_e_ from 0.75 mM to 3.5 mM induced a decrease of about 80% [90]. A close examination of the HC structure revealed that the negatively charged residue at position 50 (D50) of hCx26 is critical for HC gating and, together with the E47, might directly interact with Ca^2+^ ions to induce occlusion of the pore [73,78,91]. The D50 and E47 residues were proposed to play a role in stabilizing the HC open state in low-Ca^2+^ conditions, forming salt bridges with the K61 and R75-R184 residues, respectively. Bridge disruption at high [Ca^2+^]_e_ would destabilize the open state, thus facilitating HC closure. The conserved glycine at position 45 of hCx26, hCx30, hCx32, and hCx43 HCs was also found to be an integral part of the [Ca^2+^]_e_ sensor [92].

**Cx32:** In *Xenopus* oocytes expressing hCx32 HCs, HC currents ranged from a maximum at 0.5 mM [Ca^2+^]_e_ to a minimum value at 5 mM [Ca^2+^]_e_, with an EC50 around 1.3 mM [72]. This very high EC50 value might be due to the application of a strong (+80 mV) voltage stimulation that favoured the opening of Cx32 HCs during the current recording. The effect of voltage on the HC current dependence on [Ca^2+^]_e_ is well documented for another connexin (Cx46), whose EC50 shifted from 0.08 mM [Ca^2+^]_e_ at values ≤ −20 mV to 0.5 mM [Ca^2+^]_e_ at +20 mV [93]. Based on molecular dynamics simulations [94], this EC50 shift might be simply explained by a change in the HC binding affinity to Ca^2+^. Two Asp residues, D169 and D178, were found to be implicated in the Ca^2+^-induced blockage and conductance properties of hCx32 HCs [72]. The characteristic transitions attributable to the fast gate between a main open state of high conductance (90 pS) and a residual open state (18 pS) only occurred at low [Ca^2+^]_e_ and upon hyperpolarization. Gomez-Hernandez et al. [72] also found that substitution of Ca^2+^ in the external solution with other divalent cations inhibited hCx32 HC activation. The potential of different divalent cations to induce such a block followed the sequence: Cd^2+^ > Co^2+^ ≈ Ca^2+^ > Mg^2+^ > Ba^2+^.

**Cx37:** In *Xenopus* oocytes expressing hCx37 HCs, the HC current dependent on [Ca^2+^]_e_ ranged from a maximum at 0.02 mM [Ca^2+^]_e_ to a minimum value at 1 mM [Ca^2+^]_e_, with an EC50 of about 0.1 mM [79]. In rat brain endothelial cells (RBE4) expressing endogenous Cx37, as well as in HeLa cells expressing exogenous hCx37, dye uptake and release through Cx37 HCs were strongly inhibited by the GAP27 peptide, and single-channel electrophysiological studies indicated that GAP27 inhibits unitary HC currents [44].

**Cx43:** In rat Novikoff hepatoma cells expressing endogenous Cx43, a reduction in extracellular Ca^2+^ but not Mg^2+^ was a key factor for HC opening and dye uptake. An increased uptake started at 1 mM [Ca^2+^]_e_, reaching a maximal level at 10 μM [Ca^2+^]_e_ [63]. In *Xenopus* oocytes expressing hCx43, lowering the external divalent cation concentration (Ca^2+^ and Mg^2+^ free) decreased the resting potential and the input resistance. Both parameters recovered their initial values upon restoring the [Ca^2+^]_e_ to normal millimolar values [95]. In HeLa cells expressing rCx43, HC opening increased only modestly at positive potentials in zero Ca^2+^ or zero Ca^2+^-EGTA solutions [59].

**Cx45:** In HeLa cells expressing mouse Cx45 (mCx45), the HC current dependency on [Ca^2+^]_e_ was a combination of two Hill equations with different sensitivities (*K*_1_ = 0.66 μM and *K*_2_ = 216 μM) and a very low EC50 (~1 μM) [82]. It may be argued that the dual sensitivity reflects the coexistence of both the exogenous mCx45 and the endogenous hCx45 [96].

**Cx46:** In *Xenopus* oocytes, rCx46 HCs have a slightly lower apparent affinity for Ca^2+^ than hCx26 HCs (*K*_D_ 0.6 mM vs. 0.33 mM) [73]. Regarding Ca^2+^ sensitivity, the E48 and D51 residues of Cx46 played a role like the analogous residues E47 and D50 in Cx26. In the same cellular system [83], L35 also appeared as an important residue for HC closure by Ca^2+^, and single HC recordings suggested that divalent cations act as stabilizers of the fully closed conformation rather than as gating particles [97]. In *Xenopus* oocytes expressing hCx46, lowering [Ca^2+^]_e_ from 0.1 mM to 0.01 mM increased the HC current by approximately 150% at +20 mV, whereas increasing [Ca^2+^]_e_ from 0.1 mM to 1 mM induced a decrease of about 80% [71]. Like other connexins, a high [Mg^2+^]_e_ also acted as a blocker but to a much lower extent. The experiments by Ebihara et al. [71] applying sequential combinations of different [Ca^2+^]_e_ and [Mg^2+^]_e_ values suggested that there are at least two distinct binding sites for divalent cations that have different relative affinities for Ca^2+^ and Mg^2+^ and modulate different steps in the gating process.

**Cx50:** In HeLa cells pre-loaded with Lucifer Yellow (LY) dye and expressing hCx50, removal of [Ca^2+^]_e_ (2 mM) increased the rate of dye leakage and activated a voltage-dependent outward transmembrane current [84]. Notably, Cx50 and Cx46 share very similar amino acid sequences and HC sensitivity to [Ca^2+^]_e_. In *Xenopus* oocytes expressing mCx50 or rCx46, [Ca^2+^]_e_ shifted the I-V curve of Cx46 but not that of Cx50 HCs [98]. Cx50 HCs are also much more sensitive to external pH than Cx46 HCs. Interestingly, the replacement of extracellular Na^+^ with K^+^ or other monovalent cations while maintaining a constant high [Ca^2+^]_e_ resulted in 10-fold potentiation of mCx50 HC currents, which reversed upon restoring a normal Na^+^ concentration [70]. In contrast, rCx46 HCs exhibited a modest increase upon substituting Na^+^ with K^+^. The primary effect of K^+^ appeared to be a reduction in the ability of Ca^2+^, as well as other divalent cations, to close Cx50 HCs due to the specific connexin sequence.

## 3. Cytosolic Ca^2+^ Regulation of Connexin HCs

The [Ca^2+^]_c_ of most eukaryotic cells is maintained at ~100 nM in resting conditions, a value 10,000-fold lower than the extracellular concentration [99]. Rapid variations in [Ca^2+^]_c_ in the nanomolar or micromolar range, triggered by different extracellular and intracellular stimuli, are sufficient to activate and control several cellular mechanisms alone, such as secretion, gene expression, muscle contraction, metabolism, and also connexin channel gating [72,100,101]. The increase in [Ca^2+^]_c_ typically results from either the influx of extracellular Ca^2+^ via plasma membrane Ca^2+^ channels or the release of Ca^2+^ from internal stores via InsP3Rs and ryanodine receptors (RyRs). Connexin HCs that show an increased open probability upon [Ca^2+^]_c_ variation include Cx26 [102], Cx30 [60], Cx32 [13], Cx43 [75], Cx45 [103], Cx46 [85] (Table 1), and most likely also Cx37 [44] and Cx40 [16]. In the organ of Corti, ATP release through Cx26 and Cx30 HCs is triggered by spontaneous [Ca^2+^]_c_ oscillations in supporting cells and contributes to the propagation of the intercellular Ca^2+^ signalling that is responsible for synaptic refinement [8,87]. In astrocytes, a migratory phenotype is acquired under pro-inflammatory conditions by a [Ca^2+^]_c_-dependent opening of Cx43 HCs, followed by release of ATP, activation of the P2X7 receptor, and Ca^2+^ influx [104]. Retinal pigment epithelium (RPE) cells were found to release ATP through Cx43 HCs that increased proliferation and stimulated DNA synthesis in neural retinal progenitor cells [105]. Upon mechanical stress, an increased [Ca^2+^]_c_ elicited by the activation of Piezo1 channels opens Cx43 HCs through PI3K-Akt, which in turn leads to bone anabolic function [42]. Furthermore, NAD^+^, prostaglandin E2, and ATP release by Cx43 HCs in response to oxidative stress was shown to serve as a protective mechanism for osteocytes, which may accumulate reactive oxygen species with skeletal aging [39].

Specific information about the dependency of the HC gating on [Ca^2+^]_c_ is available for several connexin isoforms, as detailed in the following.

**Cx26:** In HeLa cells expressing rCx26, a [Ca^2+^]_c_ increase mediated by linoleic acid (LA) appeared to be essential to enhance Cx26 HC activity [102]. The synthetic Ca^2+^ chelator BAPTA and PI3K/Akt inhibitors were found to reduce ethidium (Etd) bromide uptake through Cx26 HCs, suggesting that the LA effect was mediated by the increase in [Ca^2+^]_c_ and activation of the PI3K/Akt-dependent pathway. Notably, an LA-mediated [Ca^2+^]_c_ increase stimulated Etd uptake also through mCx32, mCx43, and mCx45 HCs, while drastically reducing rCx26 GJ-mediated dye coupling [102]. In mouse cochlear organotypic cultures, focal InsP3 photoliberation elicited a [Ca^2+^]_c_ response in the irradiated supporting cells that peaked at around 500 nM and spread radially to several orders of unstimulated cells [8]. mCx26 and mCx30 HCs contributed to the propagation of this intercellular Ca^2+^ wave by releasing ATP, without the contribution of P2X7R or pannexin 1. ATP release was not observed in Cx26 KO or Cx30 KO cultures upon a [Ca^2+^]_c_ increase triggered by InsP3 photoliberation.

**Cx32:** In bladder cancer epithelial cells (ECV304) and C6 glioma cells expressing hCx32, Leybaert and co-workers [13] showed that (i) an InsP3-mediated increase in [Ca^2+^]_c_ is sufficient to trigger the opening of Cx32 HCs, promoting dye uptake and ATP release, and (ii) the HC open probability has a bell-shaped dependency peaking at 500 nM [Ca^2+^]_c_. Both small and large Ca^2+^ stimuli were ineffective in opening Cx32 HCs, and only [Ca^2+^]_c_ changes between 200 nM and 1000 nM were successful. Cytosolic photorelease of Ca^2+^ from *o*-nitrophenyl EGTA (NP-EGTA) or activation of Ca^2+^ influx by the A23187 ionophore also triggered ATP release in a dose-dependent manner, with ATP responses disappearing at stronger stimulation [13]. In single HeLa cells expressing hCx32, a local puff of extracellular ATP or histamine stimulated a [Ca^2+^]_c_ increase that triggered the opening of Cx32 HCs held at a −20 mV transmembrane potential [74]. The temporal dynamics of HC opening and closure were quantified in terms of membrane conductance variations with an exponential behaviour with rise (opening) and decay (closure) times of around 1 s.

**Cx43:** In C6 glioma and HeLa cells stably transfected with Cx43, the application of different concentrations of the Ca^2+^ ionophore 4-Br-A23187 (calcimycin) induced [Ca^2+^]_c_ transients and ATP release with a bell-shaped dependence on [Ca^2+^]_c_ which was maximal at ~500 nM [75]. Inhibition of ATP release was achieved in a concentration-dependent way by both GAP26 and GAP27, two peptides that mimic a short sequence on the first and second extracellular loops of Cx43, respectively. The peptide GAP19, which mimics a short sequence of the Cx43 cytoplasmic loop (CL), also inhibited [Ca^2+^]_c_-triggered ATP release in a concentration-dependent manner without affecting the GJ conductance and dye exchange [106]. In HeLa cells, an elevation in [Ca^2+^]_c_ from 50 to 200–500 nM in the absence of an electrical trigger was ineffective in opening single Cx43 HCs, but lowered the voltage threshold for HC opening by ~15 mV and potentiated the unitary HC current [107]. The HCs closed with a further elevation in [Ca^2+^]_c_ to 1 μM, exhibiting a bell-shaped dependence of the open probability on [Ca^2+^]_c_ peaking at 500 nM and that was very similar to the previous indirect measurement for Cx43 expressed in intact cells [75]. In isolated pig ventricular cardiomyocytes, [Ca^2+^]_c_ elevation to 500 nM potentiated the HC current, which was inhibited by GAP26 and GAP27. A similar study was performed in astrocytes of the prefrontal cortex of newborn mice, where mCx43 HC activity was found to be dependent on [Ca^2+^]_c_ and associated with D-serine release [50]. In rat brain endothelial cell lines expressing rCx43, single-cell photoactivation of InsP3 triggered an intercellular purinergic signalling which is [Ca^2+^]_c_-dependent and can be blocked by GAP26 [108]. The opening of Cx43 HCs was evoked by spontaneous elevations in [Ca^2+^]_c_ during Ca^2+^ waves in trigger cells of the chick RPE and was blocked by GAP26 [105]. The concept of trigger cells was demonstrated by luminometric extracellular ATP imaging, which showed that ATP is released as a burst from a point source of the cell [109]. Interestingly, Cx43 HC opening induced by oxidative stress in osteocytes was inhibited by the depletion in [Ca^2+^]_c_ with BAPTA-AM, suggesting that [Ca^2+^]_c_ triggers the activity of mCx43 HCs [41]. Conversely, blockade of HCs with a Cx43 antibody did not affect [Ca^2+^]_c_ [39].

**Cx45:** In HeLa cells expressing mCx45, HC openings were triggered at 100 nM [Ca^2+^]_c_ in the patch pipette [82]. In the same expression system, a progressive [Ca^2+^]_c_ rise stimulated by the application of 2.5 μM calcimycin increased Etd uptake within the cell [103]. The non-specific HC blocker La^3+^ significantly reduced this uptake.

**Cx46:** In HeLa cells stably transfected with hCx46, a [Ca^2+^]_c_ transient mediated by 2.5 μM of the Ca^2+^ ionophore ionomycin led to a dramatic increase in Etd uptake by Cx46 HCs [85]. Replacement of extracellular Na^+^ with K^+^ led to cell depolarization but reduced Etd uptake.

## 4. Pathological Alterations of HC Gating by Ca^2+^

Lost or abnormal HC activity at the plasma membrane has been associated with inflammatory conditions [110,111] and inherited diseases, like muscular dystrophy [112], syndromic and non-syndromic deafness [113], keratitis and hystrix-like ichthyosis deafness (KID/HID) syndrome [114], the X-linked form of Charcot–Marie–Tooth neuropathy (CMT1X) [115], oculodentodigital dysplasia (ODDD) [116], keratoderma–hypotrichosis–leukonychia totalis syndrome (KHLS) [117], erythrokeratodermia variabilis (EKV) [118], and congenital cataracts [119]. A single amino acid substitution in the connexin sequence can severely affect the correct HC function, leading to uncontrolled ionic leakage and release of molecules altering extracellular signalling pathways and potentially toxic for neighboring cells [120,121,122,123,124]. The biophysical properties altered in mutant HCs expressed at the plasma membrane may relate to their density, the pore selective permeability, or the gating mechanisms, including Ca^2+^-dependent regulation [73]. Several pathological mutations have been linked to altered sensitivity to extracellular and cytosolic Ca^2+^ ions, resulting in changes in the normal HC activity (Table 2). As a general criterion, the sensitivity to Ca^2+^ can be considered altered when an HC defect is present in normal [Ca^2+^]_e_, or there is a rightward or leftward shift in the HC current dependence on [Ca^2+^]_e_. Some mutations lack a clear link between HC dysfunction and Ca^2+^ deregulation. The mere observation of an increased HC activity in zero [Ca^2+^]_e_ solution, which shifts the HC to the fully open state, can also be attributed to alteration of other HC properties, such as pore permeability or voltage-dependent gating.

Mutations of the *GJB2* gene, which encodes Cx26, were linked to sensorineural hearing loss, keratitis, and severe skin lesions [125,126]. In particular, G11E, G12R, N14K, N14Y, I30N, A40V, G45E, E47Q, D50N/Y/A, and A88V mutations were associated with a reduced HC closure by [Ca^2+^]_e_ or an increased permeability to Ca^2+^ ions, which resulted in leaky HCs and compromised keratinocyte viability in vitro [78,123,127,128,129,130,131,132,133,134]. Instead, S17F, N54K, R75W, and S183F pathological mutations were associated with a decreased HC activity [123,129,130,135]. Examination of the skin of KID transgenic mice showed alterations in the epidermal [Ca^2+^]_e_ gradient that were correlated with altered lipid secretion and defects in the epidermal water barrier [133,136]. Negatively charged residues (D46, E47, Q48, D50, K61, R184) lining the Cx26 HC pore are critical for the gating dependency on [Ca^2+^]_e_, but they do not form the HC gate [73,137].

**Table 2:** Impact of connexin mutations on the HC functionality under different [Ca^2+^]_e_ and [Ca^2+^]_c_ conditions. Normal [Ca^2+^]_e_ solution refers to an extracellular solution with a Ca^2+^ concentration in the mM range (typically 2–5 mM), whereas a [Ca^2+^]_e_-free solution is prepared without Ca^2+^ or with Ca^2+^ buffered by EGTA. Abbreviations: connexin (Cx), N-terminus (NT), transmembrane domain (TM), extracellular loop (EL), cytoplasmic loop (CL), C-terminus (CT); erythrokeratodermia variabilis (EKV), keratitis ichthyosis deafness (KID) syndrome, keratoderma–hypotrichosis–leukonychia totalis syndrome (KHLS), oculodentodigital dysplasia (ODDD).

Cx Isoform	Mutation	Cx Domain	Mutant HC Defective Properties	Linked Disease
**Cx26**	**G11E**	NT	Increased Ca^2+^ leakage in normal [Ca^2+^]_e_ [127].	KID syndrome
	**G12R**	NT	Increased dye uptake in both normal and [Ca^2+^]_e_-free solutions_,_ halted [Ca^2+^]_e_-dependent deactivation kinetics, increased Ca^2+^ leakage [123,128,129].	Syndromic deafness
	**G12V**	NT	Increased dye uptake in [Ca^2+^]_e_-free solution [129].	Non-syndromic deafness
	**N14K**	NT	Reduced [Ca^2+^]_e_ sensitivity leading to increased HC currents and slowed deactivation kinetics, increased Ca^2+^ leakage [123,130,131].	Clouston syndrome/KID syndrome
	**N14Y**	NT	Increased dye uptake and HC currents in both normal and [Ca^2+^]_e_-free solutions, increased Ca^2+^ leakage [129].	Syndromic deafness
	**S17F**	NT	Decreased dye uptake in both normal and [Ca^2+^]_e_-free solutions, leaky HCs when co-expressed with Cx30 [123,129,138].	KID syndrome
	**I30N**	TM1	Increased dye uptake in both normal and [Ca^2+^]_e_-free solutions, increased Ca^2+^ leakage [132].	KID syndrome
	**V37I**	TM1	Abolished dye uptake in [Ca^2+^]_e_-free solution [139].	Deafness
	**A40G**	TM1	Abolished dye uptake in [Ca^2+^]_e_-free solution [139].	Deafness
	**A40V**	TM1	Reduced [Ca^2+^]_e_ sensitivity leading to increased HC currents [133].	KID syndrome
	**G45E**	EL1	Increased dye uptake at a normal [Ca^2+^]_e_ and a reduced [Ca^2+^]_e_ sensitivity, leading to increased HC currents and Ca^2+^ leakage [133,140].	KID syndrome
	**D46C**	EL1	Halted [Ca^2+^]_e_-dependent deactivation kinetics [73].	No association
	E47K	EL1	Halted dye uptake in [Ca^2+^]_e_-free solution [140].	Deafness
	**E47Q**	EL1	Reduced [Ca^2+^]_e_ sensitivity, leading to increased HC currents and halted deactivation kinetics [73].	No association
	**Q48A**	EL1	Increased [Ca^2+^]_e_ sensitivity, leading to decreased HC currents and reduced deactivation kinetics [137].	No association
	**D50A**	EL1	Reduced [Ca^2+^]_e_ sensitivity, leading to increased HC currents and halted deactivation kinetics [134,137].	KID syndrome
	**D50N**	EL1	Reduced [Ca^2+^]_e_ sensitivity, leading to increased HC currents and halted deactivation kinetics [73,78,123,127,130].	KID syndrome
	**D50Y**	EL1	Increased dye uptake in both normal and [Ca^2+^]_e_-free solutions, halted [Ca^2+^]_e_-dependent deactivation kinetics, increased Ca^2+^ leakage [78,132].	KID syndrome
	**N54K**	EL1	Decreased dye uptake in [Ca^2+^]_e_-free solution [130].	Bart–Pumphrey syndrome
	**R75W**	TM2	Decreased HC currents in both normal and [Ca^2+^]_e_-free solutions [135].	Deafness
	**A88V**	TM2	Increased HC currents in [Ca^2+^]_e_-free solution [134].	KID syndrome
	**S183F**	EL2	Halted dye uptake in [Ca^2+^]_e_-free solution [130].	Palmoplantar keratoderma and hearing loss
	**R184K**	EL2	Reduced [Ca^2+^]_e_ sensitivity leading to both increased HC currents and increased deactivation kinetics [73].	No association
**Cx30**	**G11R**	NT	Increased ATP release in normal [Ca^2+^]_e_ solution [141].	Clouston syndrome
	**G45E**	EL1	Reduced [Ca^2+^]_e_ sensitivity, leading to dye uptake in both normal and [Ca^2+^]_e_-free solutions [92].	No association
	**A88V**	TM2	Increased ATP release in both normal and [Ca^2+^]_e_-free solutions, increased leakage of Ca^2+^ and other ions [58,141].	Clouston syndrome
**Cx30.3**	**G12D**	NT	Increased dye uptake in [Ca^2+^]_e_-free solution [118].	EKV
	**T85P**	TM2	Increased dye uptake in [Ca^2+^]_e_-free solution [118].	EKV
	**F189Y**	TM4	Increased dye uptake in both normal and [Ca^2+^]_e_-free solutions [118].	EKV
**Cx32**	**G45E**	EL1	Increased dye uptake in [Ca^2+^]_e_-free solution [92].	No association
	**S85C**	TM2	Increased HC current in normal [Ca^2+^]_e_ solution [142].	CMT1X
	**D169N**	EL2	Reduced [Ca^2+^]_e_ sensitivity, leading to increased HC currents [72].	No association
	**D178N**	EL2	Reduced [Ca^2+^]_e_ sensitivity, leading to increased HC currents [72].	No association
	**D178Y**	EL2	Reduced [Ca^2+^]_e_ sensitivity, leading to increased HC currents [72].	CMT1X
	**R220X**	CT	Reduced [Ca^2+^]_c_ sensitivity, leading to halted HC current [74].	CMT1X
**Cx43**	**L7V**	NT	Increased ATP release in normal [Ca^2+^]_e_ solution [116].	ODDD
	**G8V**	NT	Increased HC current in [Ca^2+^]_e_-free solution [143].	KHLS
	**Y17S**	NT	Decreased dye uptake in [Ca^2+^]_e_-free solution [144].	ODDD
	**G21R**	TM1	Decreased dye uptake in [Ca^2+^]_e_-free solution [144].	ODDD
	**I31M**	TM1	Increased ATP release in [Ca^2+^]_e_-free solution [122].	ODDD
	**A40V**	TM1	Decreased dye uptake in [Ca^2+^]_e_-free solution [144].	ODDD
	**A44V**	TM1	Increased HC current in [Ca^2+^]_e_-free solution [143].	EKV
	**G45E**	EL1	Increased dye uptake in [Ca^2+^]_e_-free solution [92].	No association
	**F52dup**	EL1	Decreased dye uptake in [Ca^2+^]_e_-free solution [144].	ODDD
	**L90V**	TM2	Decreased dye uptake in [Ca^2+^]_e_-free solution [144].	ODDD
	**I130T**	CL	Decreased dye uptake in [Ca^2+^]_e_-free solution [144].	ODDD
	**G138R**	CL	Increased ATP release in [Ca^2+^]_e_-free solution [122].	ODDD
	**G143S**	CL	Increased ATP release in [Ca^2+^]_e_-free solution [122].	ODDD
	**E227D**	CT	Increased HC current in [Ca^2+^]_e_-free solution [143].	EKV
	**M239X**	CT	Abolished ATP release in [Ca^2+^]_e_-free solution and upon increased [Ca^2+^]_c_ [145].	No association
**Cx46**	**D47N**	EL1	Increased [Ca^2+^]_e_ sensitivity, leading to decreased HC currents [73].	No association
	**E48Q**	EL1	Reduced [Ca^2+^]_e_ sensitivity, leading to increased HC currents [73].	No association
	**D51N**	EL1	Reduced [Ca^2+^]_e_ sensitivity, leading to increased HC currents [73].	No association
	**N63S**	EL1	Abolished HC currents that were restored in [Ca^2+^]_e_-free solution [146].	Congenital cataracts
	**G143R**	CL	Increased dye uptake in both normal and [Ca^2+^]_e_-free solutions, decreased dye uptake upon increased [Ca^2+^]_c_ [85,147].	Congenital cataracts

Since mutations in the *GJB1* gene that encodes Cx32 were first reported in 1993 [148], more than 450 different mutations associated with the X-linked dominant form of CMT (CMT1X) neuropathy have been discovered [115]. In the PNS, Cx32 was found to selectively be expressed in non-compact myelin of Schwann cells, but its physiological role is still up for debate. The D178Y mutation in the second extracellular loop of Cx32 affects an Asp-178 residue that induces a complete [Ca^2+^]_e_ deregulation of the HC activity [72]. Cx32 HCs carrying a pathological CT domain truncation (R220X) fail to open in response to a canonical InsP3-mediated signal transduction cascade that elevates the [Ca^2+^]_c_ [74]. Interestingly, the gating function of Cx32-R220X HCs was restored by both the intracellular and extracellular application of the peptide GAP24 that mimics the Cx32 CL.

More than 70 dominant mutations, mostly autosomal, of the *GJA1* gene that encodes Cx43, were linked to ODDD, a development disorder characterized by craniofacial and limb disorders [149]. Over one-third of the mutations are localized in the CL of Cx43. In stable cell lines expressing enhanced yellow fluorescent protein (eYFP)-tagged hCx43 [144], propidium iodide uptake experiments at a low [Ca^2+^]_e,_ demonstrated that Y17S, G21R, A40V, F52dup, L90V, and I130T mutations are associated with a reduced HC function compared with the wild-type Cx43. Instead, HeLa cells expressing Cx43 carrying ODDD mutations I31M, G138R, and G143S displayed an altered HC function with increased ATP release under zero-[Ca^2+^]_e_ conditions [122]. In patient-derived fibroblasts, the ATP defect of G138R and G143S HCs was not significant, whereas the L7V mutation was found to be leaky at normal [Ca^2+^]_e_ values [116]. The downregulation of L7V HCs found in patients’ fibroblasts was proposed as a protective mechanism against cytotoxicity.

Mutations in the *GJA3* and *GJA8* genes coding for Cx46 and Cx50, respectively, are a major cause of congenital cataracts [150]. More than 40 cataract-associated mutations are located in transmembrane and extracellular loops of Cx46. Instead, the pathological mutation G143R is located in the CL of Cx46, increasing HC leakage in resting conditions and reducing HC opening upon [Ca^2+^]_c_ stimulation by ionomycin [85,147]. E47 and D50 residues in Cx26 correspond to residues E48 and D51 in Cx46. In particular, mutations E47Q and D50N/Y/A in Cx26 and E48Q and D51N in Cx46 decrease the [Ca^2+^]_e_ sensitivity (i.e., leaky HCs), while Q48A and D47N mutations increase the sensitivity in Cx26 and Cx46, respectively [73,78,137].

## 5. Discussion

Ca^2+^ ions are key regulators of connexin HC functionality in health and disease. The four orders of magnitude difference in gating sensitivity to Ca^2+^ between the extracellular and intracellular HC domains suggests the existence of at least two different Ca^2+^ sensors. Under cell resting conditions, the synergistic action of the two gates, in combination with the transmembrane voltage, keeps this relatively large and unselective pore fully closed, thus preventing cytotoxic ionic leakage. The fact that both an increased and a decreased HC Ca^2+^ sensitivity is linked to disease suggests that Ca^2+^ in healthy cells finely regulates normal HC function. While [Ca^2+^]_e_ is a relatively stable physiological parameter dependent only on the cell layer, compartment, and organ, [Ca^2+^]_c_ can change rapidly, thus triggering sudden HC opening and release of extracellular bursts of messenger molecules involved in paracrine signalling.

There is a general consensus on the gating by [Ca^2+^]_e_ through the direct binding of Ca^2+^ ions to specific extracellular residues of the six connexins forming the HC. This Ca^2+^ ring was proposed to act as an electrostatic occlusion and a stabilizer of the closed HC conformation [93,94]. Accessibility studies and numerical simulations suggest that the electrostatic effect does not hinder the access of ions or small molecules to positions deeper into the pore [78,97,151], whereas this conclusion is not generally shared [94,152]. Fitting electrophysiological experiments with Cx46 HCs by a model allosterically coupling Ca^2+^ binding and voltage sensing indicates that Ca^2+^ ions act like stabilizers of the closed HC conformation [93]. Coarse-grained molecular dynamics simulations of the Cx26 HC confirmed that Ca^2+^ coordination within the extracellular vestibule inhibits the transition to a wider pore state that would favour permeation [153]. This transition was not found in the Cx31.3 HC structure, which did not significantly change in the presence or absence of Ca^2+^ ions [154]. In single Cx32 HCs, a high [Ca^2+^]_e_ inhibited transitions from the closed to the fully open state in both depolarization and hyperpolarization conditions, thus allowing only transitions to a residual substate [72].

The model by Pinto et al. [93] considers only [Ca^2+^]_e_ and voltage as key variables of HC opening, without including the gating by [Ca^2+^]_c_ or other cytosolic chemical gating mechanisms. Indeed, in connexin isoforms such as Cx32 and Cx43, the voltage alone can trigger HC opening only at unphysiological values ≥ +40 mV [59,72,74,107]. Instead, a physiological [Ca^2+^]_c_ rise alone triggered by InsP_3_ can stimulate HC opening [74,155] while dramatically increasing the voltage sensitivity [107].

**Figure 2:**
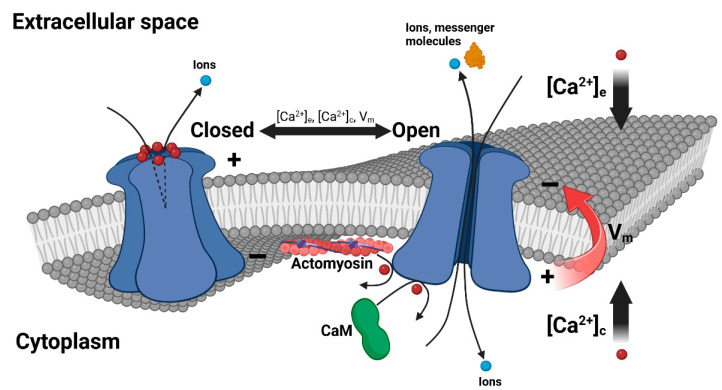
Ca^2+^ regulation of connexin HCs. Extracellular and cytosolic Ca^2+^ ions, in combination with the transmembrane voltage (V_m_), finely regulate the molecular fluxes across the connexon pore. In healthy cells, this synergistic action allows the release of extracellular bursts of messenger molecules while preventing cytotoxic ionic leakage. An extracellular ring of Ca^2+^ ions stabilizes the HC closed conformation, while CaM and the actomyosin contractile system modulate the HC opening and closure mechanisms, respectively.

The molecular mechanisms underlying the exquisite regulation of connexin HCs by nanomolar variations in [Ca^2+^]_c_ are still under debate. The interaction of the CL with the CT domain and calmodulin (CaM) was found to control the gating of both connexin GJs [156,157,158,159] and HCs [13,74,145,160]. In the pathological G143R mutation of Cx46, HC dysfunction was attributed to an increased interaction of the CL with the CT domain and CaM, possibly caused by the loss of the CL α-helical structure [85]. Lack of the CT domain in Cx32 [74] and Cx43 [145] caused the HC to fail to open in response to a [Ca^2+^]_c_ increase. The opening was restored upon application of peptides (GAP24 and TAT–Cx43CT10, respectively) that are able to interact with the CL domain. Leybaert and coworkers [161] proposed that loss of the CL-CT domain interaction by a Ca^2+^-dependent activation of the actomyosin contractile system underlies the Cx43 HC closure mechanism upon [Ca^2+^]_c_ overload (above 500 nM). Peracchia et al. [159] have proposed that CaM acts both as a Ca^2+^ sensor and a cork at the cytoplasmic mouth of the connexon. This hypothesis is supported by the findings that CaM co-localizes with GJs [85,162] and has a highly Ca^2+^-dependent affinity to all three intracellular connexin domains [163,164]. Furthermore, HC oligomerization and its opening can be prevented by the W7 CaM inhibitor [13,75,84,165] (Figure 2).

It is noteworthy to mention that the pathological G12R and N14K mutations of Cx26 both reduce the affinity of the NT to CaM [128,131]. In human and murine fibroblasts carrying the pathological G12S and S26L mutations of Cx32, an increased CaM-dependent protein kinase II (CaMKII) activity was linked to the CMT1X motor phenotype, mitotic instability, and HC dysfunction [166]. The defects were partially recovered by a CaMKII inhibitor (KN93), supporting the notion that a CaM-dependent pathway controls the HC gating by [Ca^2+^]_c_ [13,75,167].

Overall, further investigation is needed to clarify the structural and chemical modifications of connexin HCs during opening by [Ca^2+^]_e_ and [Ca^2+^]_c_ variations. A more complete model accounting for Ca^2+^ and transmembrane voltage changes will undoubtedly improve our interpretation of the experimental results performed under physiological conditions. A model of this kind could open unprecedented opportunities for the development of therapeutic compounds that target specific HC dysfunctions. Recently, novel classes of molecules have been developed that directly interact with connexin HCs, including connexin mimetic peptides [168], anti-connexin antibodies [169], and aminoglycosides without antibiotic activity [170]. Although the mechanism of action for some of these molecules is not yet fully understood, their increased selectivity for specific connexin isoforms, combined with their reduced toxicity, makes them promising candidates for clinical application.

## Figures and Tables

**Table 1:** HC activity regulation by [Ca^2+^]_e_ and [Ca^2+^]_c_. When available, the half maximal effective concentration (EC50) was reported. [Ca^2+^]_e_-free solution refers to an extracellular solution prepared without Ca^2+^ or with Ca^2+^ buffered by EGTA.

Cx Isoform	HC Regulation by [Ca^2+^]_e_	HC Regulation by [Ca^2+^]_c_
**Cx26**	Current ranging from a maximum at 0.01 mM to a minimum value at 10 mM [Ca^2+^]_e_, with an EC50 around 0.25 mM [78].	ATP release increases with [Ca^2+^]_c_ around 500 nM [8].
**Cx30**	Current increases in [Ca^2+^]_e_-free solution [60].	ATP release and dye uptake increases with [Ca^2+^]_c_ around 500 nM [8].
**Cx30.2/31.3**	ATP release increases in [Ca^2+^]_e_-free solution [12].	Information not available.
**Cx32**	Current ranging from a maximum at 0.5 mM to a minimum value at 5 mM [Ca^2+^]_e_, with an EC50 around 1.3 mM [72].	Bell-shaped dependence of ATP release on [Ca^2+^]_c_, peaking at 500 nM [Ca^2+^]_c_ [13].
**Cx37**	Current ranging from a maximum at 0.02 mM to a minimum value at 1 mM [Ca^2+^]_e_, with an EC50 around 0.1 mM [79].	Information not available.
**Cx39**	Dye uptake increases in [Ca^2+^]_e_-free solution [80].	Information not available.
**Cx40**	HC pore size increases at [Ca^2+^]_e_ < 10 μM [81].	Information not available.
**Cx43**	Dye uptake ranging from a maximum at 0.01 mM [Ca^2+^]_e_ to a minimum value at 1 mM [Ca^2+^]_e_ [63].	Bell-shaped dependence of ATP release on [Ca^2+^]_c_, peaking at 500 nM [Ca^2+^]_c_ [75].
**Cx45**	Bi-sigmoidal dependence of the current on [Ca^2+^]_e_, with EC50 around 1 μM [Ca^2+^]_e_ [82].	Current increases with [Ca^2+^]_c_ around 100 nM [82].
**Cx46**	Current ranging from a maximum at 0.01 mM to a minimum value at 1 mM [Ca^2+^]_e_ [71].	Dye uptake increases upon [Ca^2+^]_c_ elevation [85].
**Cx50**	Dye leakage increases in [Ca^2+^]_e_-free solution [84].	Information not available.
